# Supplementary material for: Phytohormones and Transcriptome Analyses Revealed the Dynamics Involved in Spikelet Abortion and Inflorescence Development in Rice
Source: Int J Mol Sci. 2022 Jul 17;23(14):7887. doi: 10.3390/ijms23147887 (PMC9324563; doi:10.3390/ijms23147887)
Supplement: Supplementary file 1 [file ijms-23-07887-s001.zip › Supplementry Tables.pdf]

Table S1. List of DEGs showing the comparison of RNA-seq and RT-qPCR expression.

Gene	Forward Primer	Reverse Primer	FC RNA-Seq	FC RT-qPCR
Os12g0637000	AGTGGAGGTGGCTGGAG	ATGTAGTGGTATCCGTTGCTG	-8.9	-3.9
Os04g0570600	TGGCATTCACATCTCAGGTC	ACCATCACTCCCCATCCT	-8.81	-4.5
Os12g0242700	GGCCAATAACCTCGTCTCATC	GCTCTTCCAATCCCTCTAGATG	-8.4	-3.6
Os12g0242700	GTGAACCACACCCTGGAG	AGAGTAGAGAGGCGGCAC	-8.4	-8.6
Os04g0178300	CAAACAGGAGACCAGAAATGC	TGAAGTGCCTTGATATGCCC	7.90	3.5
Os07g0526600	CGGGAAAGCATCAAAGACAAG	ACCCCTCTGAAATTGTAAGCTC	3.7	4.3
Os09g0368200	CATCGTCAACTCCACCCTC	CCACTCTTGTCCACTTCATCTG	7.4	3.7
Os01g0196300	CATGATCTCGGAGCGGAAG	CTCTGACAGCTTTGACTCGAG	7.2	4.3
